# Reflections on bio-based PET and plastic waste management: a responsible research and innovation approach

**DOI:** 10.1038/s41467-026-69970-4

**Published:** 2026-03-06

**Authors:** Joanne Benton, Catalina Cruañas Paniker, Brooke Wain, José I. Jiménez

**Affiliations:** 1 Department of Life Sciences, Imperial College London, London, UK; 2 Science and Solutions for a Changing Planet DTP, Imperial College London, London, UK; 3 Imperial Centre for Engineering Biology, London, UK

**Keywords:** Bioremediation, Environmental biotechnology

## Abstract

Plastics drive twin crises: persistent pollution and greenhouse gas emissions. Bio-based approaches using enzymes and microorganisms to depolymerise plastics and valorise monomers show promise but raise societal, ethical and regulatory questions central to Responsible Research and Innovation (RRI). In this Perspective, we reflect on RRI implications of bio-based plastic degradation, informed by stakeholder discussions across the plastics value chain and public engagement. We identify broad support alongside concerns about scalability, interaction with existing recycling, governance and containment of genetically modified organisms, management of additives and contaminants, and the roles of regulation and economic incentives in enabling adoption.

## Abstract (web summary)

Bio-based approaches to plastic degradation have been an intense area of research in recent years, and although they show great promise, they also raise societal, ethical and regulatory questions. Here the authors reflect on the Responsible Research and Innovation (RRI) implications of this growing field, sharing insights they have gained through engagement with stakeholders and the broader public.

## Introduction

Our everyday reliance on plastic materials, combined with often inappropriate disposal practices, has contributed to a well-documented global plastic pollution problem affecting aquatic and terrestrial environments, human health, and the climate. OECD business-as-usual projections estimate that by 2060 global plastic use will have tripled relative to 2019 levels, reaching 1231 Mt annually^1^. Over the same period, plastic waste generation is projected to rise to 1014 Mt, with an estimated 34–55 Mt leaking into the environment each year^1^. In 2019, only 9% of global plastic waste was recycled, while 19% was incinerated, nearly 50% was landfilled, and 22% was mismanaged. Although recycling rates are projected to increase modestly to 17% by 2060, incineration and landfill are still expected to dominate waste management pathways, at approximately 18% and 50%, respectively. These trends underscore the urgent need for more effective strategies to reduce the current and future impacts of plastic waste.

Given the complexity of the situation, a multifaceted approach is necessary to address the challenges associated with the disposal of plastic material once it has reached the end of its functional life. In addition to innovative recycling technologies, other important considerations include investment in waste collection and management systems, in the design of novel polymers with improved lifecycles and fundamental changes in consumer behaviour^2^. Furthermore, the OECD calls for more to be done to boost innovations that reduce the amount of virgin materials needed for plastics manufacture, extend the useful life of plastic materials and promote plastic recycling^3^. While mechanical recycling of plastic is an established technology, chemical and specifically biological innovations are in the development phase. A publication by the European Patent Office (EPO) showed that for the period 2010 to 2019, biological and chemical recycling innovations represented twice the number of international patent families (IPFs) than for mechanical recycling with 1500 IPFs involving living organisms^4^.

The potential of microorganisms and enzymes to break down plastic waste into small molecules or monomers continues to be the subject of many reviews^5–17^. Most studies focus on the biodegradation of plastics such as polyethylene terephthalate (PET), commonly found in bottles and textiles and polyurethane (PU), found in insulation materials and mattresses among many other products. This is due to the presence of hydrolysable ester bonds within the backbones of PET and ester-based PU, which are susceptible to esterase attack leading to biodegradation^18^. In contrast, the stable covalent bonds found within the backbones of plastics such as polyethylene (PE), polypropylene (PP) and polystyrene (PS) confer intrinsic resistance to enzymatic cleavage, as they lack reactive heteroatom-containing linkages that enzymes can readily target. As a result, the identification and engineering of microorganisms and enzymes capable of degrading PET and some PUs has dominated the field, whereas true depolymerisation of polyolefins remains extremely challenging and typically proceeds through oxidative, radical, or co-metabolic processes that do not regenerate the original monomers.

Despite the limitations for many polymer classes, PET has become the principal model substrate for biological plastic degradation because its ester linkages can be made accessible, particularly in amorphous or pre-treated PET^19–23^, to nucleophilic attack by hydrolases^24^. However, this accessibility is strongly influenced by the physical properties of the polymer. PET contains a substantial crystalline fraction that is recalcitrant to enzymatic hydrolysis below its glass transition temperature (~70 °C)^25–27^. As a result, efficient depolymerisation requires elevated temperatures or pre-treatment to increase chain mobility and expose ester bonds. These intrinsic constraints are shared by other hydrolysable polymers, such as polyamides (nylons) and many PUs, which often exhibit even higher glass transition temperatures or crystallinity, further limiting enzymatic attack. Importantly, these considerations also explain why biodegradation of microplastics (MPs) and textile fibres is particularly challenging, as these materials are typically encountered at low environmental temperatures (e.g. <30 °C in washing machine effluents), far below the conditions required for effective enzymatic hydrolysis.

The notable breakthrough for this field was the identification of *Piscinibacter sakaiensis*, formally known as *Ideonella sakaiensis*, a bacterium that secretes PETase and MHETase enzymes to break down PET into its constituent monomers, terephthalic acid (TA) and ethylene glycol (EG) for use as a carbon and energy source^28^. Since then, numerous bacterial and fungal cutinases have been discovered and extensively studied^29–33^, with an updated list of characterised enzymes available on the Pazy database (www.pazy.eu). However, the wild-type PETase showed limited activity, particularly on semi-crystalline PET and natural microbial degradation was slow^34^, motivating extensive efforts on isolating or engineering more efficient plastic-degrading enzymes and microorganisms. Subsequent studies have produced more efficient hydrolases, including a thermostabilised PET hydrolysing leaf-branch compost cutinase (LCC^ICCG^) engineered to operate near PET’s glass transition temperature^26^; a synergistic two-enzyme PETase-MHETase system with improved monomer turnover compared to free enzymes^35^; the polyester hydrolase PHL7 from a compost metagenome, highly active on amorphous PET films, such as those used for fruit packaging^36^; and the FAST-PETase variant capable of degrading post-consumer thermoformed PET^37^. Furthermore, these enzymes can also be expressed in thermophilic hosts, enabling hydrolysis at elevated temperatures such as for PET, where the substrate becomes more susceptible to biodegradation.

The resulting monomers released from enzymatic PET degradation, TA and EG, can be repolymerised into recycled PET (rPET), enabling true closed-loop recycling and contributing to a circular economy for polyester-based materials^26,36,37^. However, due to the lack of hydrolysable bonds, such closed-loop recycling is currently feasible only for polyester-based plastics like PET. Although recent progress in other degradable polymer classes highlights the plastic-specific nature of biocatalytic recycling, one major advance is the development of an engineered polylactic acid (PLA) depolymerase that can be embedded directly into PLA. This approach enables self-degrading mulch films^38^ and demonstrates how tailored enzyme–polymer design can allow controlled depolymerisation in situ. Similarly, the discovery of true urethanases capable of hydrolysing urethane linkages in PUs marks an important step forward, as earlier reports relied mainly on cutinases that hydrolysed only ester-containing segments^39,40^. Urethanases have the potential to release defined diamine building blocks, such as 4,4′-methylenedianiline (MDA) and toluene-2,4-diamine (TDA), in contrast to chemical hydrolysis, which produces complex mixtures requiring intensive downstream separation.

By contrast, the enzymatic or microbial depolymerisation of hydrocarbon polymers remains out of reach and despite several studies suggesting enzymatic activity against these polymers, re-evaluation has shown that the evidence is weak or unsupported, as demonstrated by Stepnov et al., who found no verifiable biodegradation of PE or PVC by the previously claimed enzymes^41^. These findings emphasise that successful biocatalytic depolymerisation depends on polymer chemistry, physical structure and engineered enzyme specificity, and that universal plastic biodegradation remains scientifically unrealistic.

**Fig. 1:**
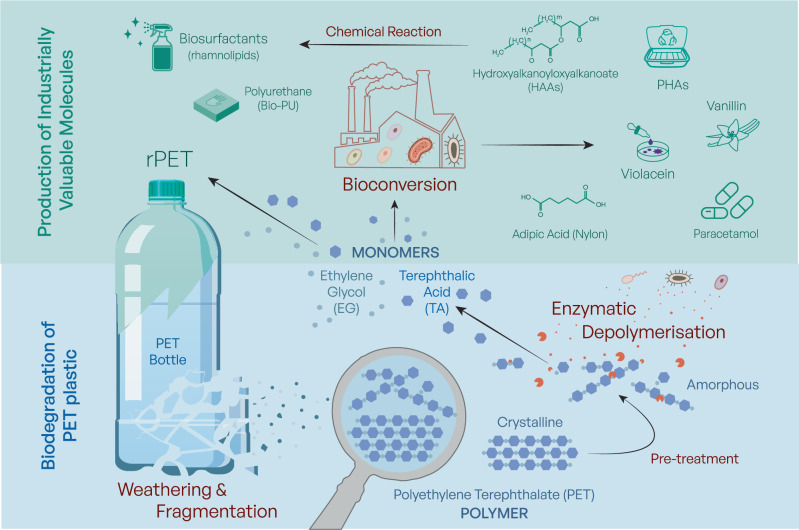
Biological management and valorisation of poly(ethylene terephthalate) (PET) plastic waste. PET plastic waste in the environment undergoes fragmentation and weathering, generating smaller particles and microplastics. PET is shown at the polymer-chain level in both crystalline and amorphous states, and crystalline PET can undergo physical or chemical pre-treatment to increase amorphous content and enhance enzymatic accessibility. Purified PET-depolymerising enzymes, or enzymes secreted during whole-cell biocatalysis, cleave the ester bonds linking the monomers, yielding terephthalic acid (TA) and ethylene glycol (EG). These monomers can be purified and repolymerised to produce recycled PET (rPET) with properties comparable to virgin PET, enabling closed-loop recycling. Alternatively, TA and EG can be biotransformed by engineered microorganisms into value-added compounds, including vanillin, violacein, paracetamol, rhamnolipids, adipic acid and hydroxyalkanoyloxyalkanoates (HAAs), or converted via chemical upgrading routes into precursors for biopolymers such as polyhydroxyalkanoates (PHAs) and other useful materials, including bio-based polyurethane (Bio-PU) derived from HAAs, supporting open-loop upcycling pathways. Note that several value-added products shown represent niche, high-value outlets and do not constitute PET-scale end markets; in most scenarios, most recovered monomers would return to polymer production, with only a fraction diverted to higher-margin chemicals.

Nonetheless, biotechnology also allows for the upcycling of plastic waste, a process known as open-loop recycling where plastics, such as PET or PU, are enzymatically degraded with the released monomers undergoing biotransformation by engineered microorganisms into higher-value chemicals. Examples include vanillin^42^, violacein^43,44^, paracetamol^45^, hydroxyalkanoyloxyalkanoate (HAA) that can be used in the synthesis of Bio-PU (bio-based poly(amide urethane) and biopolymers such as polyhydroxyalkanoate (PHA)^46–48^, illustrated in Fig. 1.

Ultimately, the goal is to develop these bio-based technologies for operation at an industrial scale; however, it must be noted that achieving this will require polymer-specific, engineered enzymes and microbes for different plastics, as biocatalytic recycling depends on the distinct chemical and physical properties of each material. For example, Carbios, in collaboration with the University of Toulouse and partners, have successfully developed an LCC-based PET depolymerisation process that has reached technology readiness level (TRL) 8 and is currently being scaled to a multi-ktonne industrial facility^26^. Additional companies targeting PET biorecycling include Samsara Eco, while other enzyme-engineering start-ups, such as Epoch Biodesign, are developing broadly applicable platforms for multiple plastic waste streams^49^.

These industrial developments, together with recent enzymatic breakthroughs in PLA and PU depolymerisation, illustrate the rapid advances in polymer-specific biocatalysis and portrays a future where biotechnological approaches will make a significant contribution to the proper management of plastic waste. To ensure these technologies are deployed responsibly and effectively, bridging gaps between scientists, industry, policymakers and the public are essential^50^. Within this context, addressing Responsible Research and Innovation (RRI) will play a significant part in funding programmes across the world, where it provides a framework for fostering openness, reflexivity and two-way engagement, enabling stakeholders and communities to articulate their insights, expectations and concerns. Rather than aiming to create a unified front, RRI emphasises inclusive dialogue and mutual learning, ensuring that the development, communication, and eventual commercialisation of biotechnological solutions align with societal values and priorities^51^.

RRI aims to connect science with society by involving stakeholders and citizens in Research and Innovation (R&I) to help ensure that outcomes are not only scientifically robust but also ethically acceptable, socially desirable and environmentally sustainable^52^. Foundational scholarship describes RRI as a framework built around anticipation, inclusion, reflexivity and responsiveness, emphasising the need to shape innovation trajectories before technologies become locked^53–55^. Within biotechnology and emerging industrial bioapplications, RRI has been applied to questions of biosafety governance, public legitimacy and the responsible design of novel bio-based systems, particularly where technologies intersect with societal values and regulatory uncertainty^56–59^. Central to RRI is therefore not simply engagement for its own sake, but an ongoing commitment to openness, transparency and critical reflection. Public engagement plays a key role in this process: it raises awareness of emerging research, creates opportunities for dialogue and helps build trust between scientists and society. By enabling people to voice questions and concerns early, public engagement supports more responsive research practices and can foster more informed, constructive attitudes toward innovation.

Previous publications have addressed technical misconceptions and challenges relating to biotechnological plastic degradation and recycling^60^, but broader engagement with stakeholders and consideration of public perception have received less attention. This Perspective addresses this gap by reflecting on themes arising from stakeholder and public engagement activities undertaken alongside the ERA CoBioTech MIPLACE project. The scientific programme of the project focused on identifying and engineering microbes and enzymes to (i) biodegrade PET and PU waste into their constituent monomers and (ii) biotransform monomers into molecules for use in the synthesis of Bio-PU, thereby contributing to a circular economy for plastics.

### Engagement activities informing this Perspective

The reflections developed in this Perspective are informed by sustained engagement with stakeholders and public audiences between June 2021 and February 2025. Over this period, we engaged with contributors across the plastics value chain, including representatives from industry (waste management, water, PE, PP, PU and PLA manufacturing, and mechanical and chemical recycling), as well as social enterprises, NGOs and academia. All engagement activities were conducted with informed consent, and no personally identifying information was collected or reported (Supplementary Note 1). Engagement primarily took the form of semi-structured conversations, initiated through email invitations and supported by a short project overview document (Supplementary Note 2) outlining the aims and scope of the MIPLACE project. Conversations typically lasted 30–60 min, with the prompts used to guide these conversations provided in Supplementary Note 3, and detailed notes were used by the author team to capture the themes, concerns and expectations raised. Further details of engagement formats are provided in Supplementary Note 2.

To complement these stakeholder discussions, we also engaged with wider public audiences through interactive formats. A 2-day workshop on microbial degradation of plastic waste for children aged 5-12 and their families was delivered at the Great Exhibition Road Festival in June 2022. Although primarily designed as an educational activity, the workshop also provided opportunities for audiences to share their views through simple attitudinal responses and written comments. In September 2022, an interactive presentation was delivered to an adult audience, typically over 50 years old, from three London boroughs. These engagements offered further insight into how bio-based approaches to plastic waste are perceived beyond professional stakeholder groups, with further details of these public-facing engagement activities provided in Supplementary Note 4 with their scope and limitations outlined in Supplementary Note 5.

**Fig. 2:**
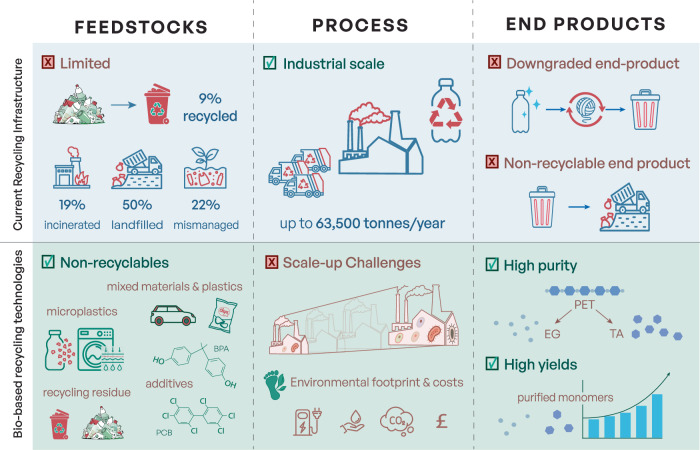
Overview of perspectives on the advantages and disadvantages of standard versus biological plastic recycling. Discussions highlighted key differences between the two approaches, including the types of plastic feedstocks required, the operational scale, the quality of the resulting products and the nature of the waste streams produced. Light blue indicates views related to the current recycling infrastructure, encompassing mechanical recycling, chemical recycling and the broader linear recycling economy. Green indicates key points related to biobased recycling technologies that employ microbes and enzymes to recover monomers. BPA bisphenol A, PCB polychlorinated biphenyls, PET poly(ethylene terephthalate), EG ethylene glycol, TA terephthalic acid.

Across these activities, there was broad support for the use of biotechnology to address plastic waste. At the same time, discussions repeatedly highlighted a need for clearer information about what bio-based approaches entail in practice, including questions around feedstock origin, scalability and the fate of end-products. As with many other bio-based approaches, there are reservations about technologies that rely on living systems, which were perceived as more difficult to control or contain. While overall responses to technologies aimed at tackling plastic pollution were positive, the interactions made us more cautious about the large-scale upcycling of PET and PU waste using biotechnological routes, pointing to gaps that would need to be addressed for future implementation (Fig. 2). The following sections draw on these reflections to discuss key challenges and opportunities for plastic biodegradation within a RRI framework.

### Unlocking new waste streams

#### Avoiding feedstock competition: the PET case study

Despite the successful scaling-up of enzymatic hydrolysis of PET by Carbios, it has been questioned whether PET is the most appropriate feedstock for biological upcycling, given the existence of efficient and economical PET-recycling technologies. As a result, this bio-based approach may initially appear to compete with well-established mechanical recycling methods, which, after decades of optimisation, operate at high efficiency and at low cost. Rather than dismissing this concern, it is important, within an RRI framing, to recognise that such questions highlight areas where clearer communication and shared understanding are needed. These discussions revealed that, while established mechanical and chemical routes recover substantial quantities of PET, they do not cover the entirety of the PET waste landscape. There is still a substantial amount of PET waste that is considered non-recyclable, due to factors such as repeated mechanical recycling or virgin PET incorporated into complex mixed materials, contaminated with chemical additives. Even with investment in collection and sorting technologies, the current infrastructure alone will not be able to scale alongside the rate at which PET waste is increasing, with the OECD predicting global PET waste to reach 62 Mt by 2060^1^.

These concerns, therefore, help identify a key communication point: that bio-based PET recycling is not intended to replace existing processes but to complement them by targeting waste streams currently excluded from conventional recycling routes. In this context, bio-based PET management would enable the recycling of larger amounts of PET, unlocking waste streams that have been traditionally overlooked by current recycling routes^61^, thereby increasing overall recycling capacity and broadening the range of materials that can be returned to circular value chains.

In addition, Extended Producer Responsibility (EPR) schemes will also increase the amount of material available for recycling^62^. These schemes hold manufacturers accountable for the cost of collecting, managing, recycling and disposing of post-consumer packaging waste^63^. EPRs also aim to incentivise the design of more recyclable products through fee eco-modulation, where fees are adjusted according to a product’s recyclability. It is also important to note that many conventional recycling processes lead to a reduction in molecular weight, requiring the addition of virgin material to maintain product performance. By contrast, full biological depolymerisation can regenerate monomers of ‘virgin-equivalent’ quality, allowing complete repolymerisation without blending and thereby contributing more effectively to increasing recycling quotas under EPR frameworks.

#### Expanding the recyclability of PU, PS, PE and PP

To date, biodegradation of plastics has mainly focused on PET, a plastic type for which several recycling technologies exist^24^. It has been noted the lack of comparable biotechnological approaches for other plastics, particularly polyolefin-derived materials, such as multilayer films and packaging. Since biological recycling remains in its infancy, developers have focused primarily on polymers that are theoretically amenable to enzymatic depolymerisation, most notably PET and certain PUs whose polyester content, as previously mentioned, provide hydrolysable ester bonds absent in polyolefins. It is anticipated that hurdles related to scaling-up, sustainability and economic feasibility will share similarities across polymer classes, so the implementation of PET biodegradation at scale will provide valuable insights for the development of biocatalytic strategies for other plastics.

However, it is important to acknowledge the current scientific limits of enzymatic plastic degradation. While progress has been made for polyester- and urethane-containing materials, there is presently no robust evidence for true enzymatic depolymerisation of polyolefins such as PE, PP or PS. These polymers possess highly inert, fully saturated carbon–carbon backbones that are not susceptible to nucleophilic attack by hydrolases or oxidoreductases. Apparent biodegradation reported in earlier studies has been re-evaluated and shown to arise from abiotic processes or methodological artefacts, as demonstrated in recent detailed analyses, including work led by Stepnov and colleagues in 2024^41^. These studies confirm that polyolefin biodegradation requires prior abiotic depolymerisation to lower-molecular-weight hydrocarbons (typically <C40) before any microbial metabolism can occur, meaning that direct enzymatic attack on the polymer chain remains unsubstantiated. In contrast, genuine progress has been achieved in other hydrolysable polymers^6,7,18,64,65^ with recent research demonstrated for PUs, with urethanases and some amidases showing clear activity on PU oligomers^66^.

Previous collaborative projects such as the Enzymatic Recycling of Polyester Plastics (Enzycle) programme (https://www.enzycle.eu) and the MIXed plastics biodegradation and UPcycling using microbial communities MIX-UP project (https://www.mix-up.eu), together with ongoing initiatives including the U.S. Department of Energy multi-organization Bio-Optimized Technologies to keep Thermoplastics out of Landfills and the Environment (BOTTLE™) consortium (https://www.bottle.org/) and the UK Research and Innovation (UKRI) Mission Hub ‘Preventing Plastic Pollution with Engineering Biology’ (P3EB) led by the University of Portsmouth (https://p3eb.org.uk/), continue to advance the frontiers of biological plastic degradation through the discovery and engineering of novel enzymes, pathways and microbial systems. While some of these programmes have explored polyolefin biodegradation, substantive progress has largely been confined to hydrolysable or partially hydrolysable polymers, where enzymatic depolymerisation is chemically feasible. This underscores that biocatalytic recycling will depend on polymer-specific enzyme engineering and that certain plastic classes are fundamentally less amenable to biological breakdown than others.

#### Targeting mixed plastics and mixed materials

Our discussions have also highlighted the possible advantage of biobased approaches for dealing with mixed plastics and mixed-material waste streams. Currently, blended plastics (combinations of polymers within a single product) or mixed materials (plastics combined with substrates such as wood, metal, glass, textiles or composites) are largely unrecyclable and typically discarded as waste.

Although it is technically feasible to separate and recycle some of the plastics present within mixed materials, such as those found in furniture or vehicles, this process can be labour-intensive, energy-demanding and often economically prohibitive^67^. However, microbial or enzymatic degradation, by contrast, could provide a mechanism to overcome the need for physical separation^5,68^. By selectively depolymerising the plastic fraction of a heterogeneous material, enzymes would hydrolyse polymer chains to monomers or oligomers that could be removed from the non-plastic fraction^69,70^. For example, Carbios’ PET hydrolase enables the selective recovery of polyester from textile blends not amenable to conventional recycling^71^. Similarly, a cutinase from *Humicola insolens* has been shown to depolymerise PET within PET/cotton mixtures to release TA, while cellulases act on the cotton fraction to generate glucose^72^. Additionally, only secreted or cell-surface-exposed enzymes can act directly on plastic substrates, as intracellular enzymes cannot access solid polymers. This is feasible for PET and other hydrolysable polymers for which secreted hydrolases exist, but it presents significant challenges for the more recalcitrant plastics such as PE, PP, PVC and PS, which would require cocktails of highly specialised oxidative or hydrolytic enzymes, none of which currently exhibit verified activity on intact polyolefin chains.

These mechanistic constraints also shape the distinction between enzyme-based and microbial approaches. Future concepts often envisage microbial consortia in which different organisms contribute distinct functions, or alternatively enzyme cocktails composed of purified hydrolases with complementary substrate specificities. Enzyme-only strategies, using purified or immobilised hydrolases, avoid the biosafety concerns associated with living organisms but require substantial protein engineering to achieve activity and stability across disparate substrates. In contrast, concepts based on microbial consortia, introduce additional considerations, including containment of engineered strains, risks of environmental release, genetic stability and unpredictable interactions within complex waste streams. As such, while mixed-plastic biodegradation is an appealing long-term goal, its practical implementation will require careful alignment of polymer chemistry, enzyme accessibility and the biological risks associated with deploying living systems.

#### Targeting plastic additives

A common concern for experts in recycling, manufacturing and regulation is the presence of chemical additives and legacy compounds in plastic polymers. Additives such as plasticisers, stabilisers and flame retardants, including halogenated compounds such as brominated or chlorinated species, as well as more recently adopted phosphorus-based flame retardants^73,74^, give plastics desirable properties but can also hinder conventional recycling operations^75^. Considering the high specificity of biochemical processes, biodegradation may offer opportunities to address plastics containing such additives; however, the interactions between additives and biological systems remain insufficiently understood. On one hand, the presence of additives might negatively affect plastic biodegradation by impairing enzymatic or microbial activity through toxic or inhibitory effects. On the other hand, certain additives may be more readily catabolised than the polymer itself, potentially altering degradation pathways or generating intermediates that influence overall process efficiency^76^. Furthermore, biological recycling could increase the sustainability of recycling plastics with additives, such as being advantageous for plastics containing flame retardants where lower processing temperatures reduce the risk of forming the corrosive or hazardous byproducts known to arise during chemical recycling of halogenated materials^77^.

In addition to additives, legacy chemicals present in plastics have been classified as substances of very high concern and persistent organic pollutants (PoPs)^75^. The presence of these chemicals in plastic waste adds another layer of complexity to the recycling process as they need to be safely removed and disposed of to prevent the contamination of new products^77^. Although early studies have begun to demonstrate enzymatic transformations of persistent pollutants, including emerging work on polyfluoroalkyl substances (e.g. PFAS)^78^, the integration of such biocatalytic detoxification steps into recycling workflows remains a significant research frontier.

Further consideration needs to be given to the implications of continuous closed-loop recycling systems. For polymers such as PET, full depolymerisation and repolymerisation enable multiple recycling cycles without the progressive loss of material quality characteristic of mechanical recycling. However, these highly efficient closed-loop processes may also increase the risk of accumulating additives, dyes, trace contaminants or persistent ‘forever chemicals’ if they are not fully removed, mineralised or degraded during processing. Ensuring that enzymatic recycling workflows incorporate robust purification or detoxification steps, whether chemical, physical or biological, will therefore be essential to prevent inadvertent concentration of harmful substances across successive cycles.

Considering these issues through an RRI lens highlights how stakeholder perspectives broaden the scope of scientific and technical concerns. While researchers often emphasise enzyme specificity, reaction conditions and process efficiency, stakeholders additionally question additive toxicity, environmental persistence, bioaccumulation risks and the governance of closed-loop systems. These insights underline the importance of integrating societal concerns into future research directions, communication strategies and innovation pathways. In doing so, RRI offers a means to anticipate and address risks early, ensure transparency in the management of hazardous additives and align emerging bio-based recycling technologies with the expectations and values of the communities they ultimately serve.

#### Targeting microplastics (MPs)

The biodegradation of MPs is drawing heightened attention. These are small plastic particles, typically <5 mm, generated either intentionally (e.g. nurdles used as industrial feedstock) or unintentionally through weathering and fragmentation of larger plastic items in the environment, as well as through routine processes such as laundry of polyester or polyamide textiles which release plastic microfibres. Once formed, MPs disperse widely across aquatic, terrestrial and atmospheric environments, where they are difficult to collect and have been associated with adverse effects on wildlife and human health^79^. One of the largest reservoirs of MPs is wastewater (WW) and subsequent sewage sludge, where MPs enter WW from domestic, industrial and urban run-off streams to then accumulate within sludge^80–83^. In several countries, the high concentration of MPs in sewage sludge has become a significant barrier to its beneficial reuse, including applications in agriculture or soil improvement and as a result, sludge that would otherwise be considered a valuable resource is instead incinerated to prevent the further release and accumulation of MPs in terrestrial environments.

Since MPs are shifting into the wider public’s concern, several health policies have been developed to prevent the accumulation of MPs in different environments. For instance, as of January 2025, France has taken action to prevent MPs from laundry seeping into the aquatic environment by introducing a requirement for new washing machines to be fitted with microfibre filters^84^, a measure currently being debated in other countries including the UK. However, the appropriate disposal of the trapped microplastic fibres is still unclear^85^. Although household waste in France and the UK is largely directed to incineration rather than landfill, inappropriate disposal or export of waste could still allow microplastic fibres to enter terrestrial environments. A bio-based approach could therefore provide a route for degrading captured fibres, whether through enzymes incorporated into the filtration system within a domestic setting or at a larger scale via enzyme- or microbe-based degradation at municipal facilities following filter collection^86^. Early studies have also demonstrated the enzymatic degradation of polyester MPs in soil^87^, highlighting the potential for targeted bio-based interventions beyond conventional waste streams.

Wastewater treatment plants (WWTPs) also receive considerable microplastic loads from sources such as run-off water, which carries MPs derived from road markings, tyre wear and fragmentation of discarded plastic litter. WWTPs rely on the use of bacteria to remove organic pollutants, specifically organic carbon, nitrogen and phosphorus, across two main phases following separation of the liquid fraction from the settled organic solids. In secondary treatment, aerobic microorganisms in activated-sludge tanks remove dissolved organic pollutants, nitrogen and phosphorus. Once the treated effluent meets regulatory standards, where the pollutant levels of the liquid waste drop below the threshold, it is discharged back into waterways. The remaining organic matter, or ‘sludge’, is then processed through anaerobic digestion, where anaerobic microbial communities stabilise the material and generate biogas. The resulting digestate can subsequently be applied to agricultural land as a soil improver or, alternatively, incinerated for energy recovery.

Due to the increasing accumulation of MPs in wastewater streams, there has been growing interest in whether bio-based interventions could be incorporated into existing WWTP process to mitigate MP release. However, despite the appeal of using biological systems within aeration tanks or anaerobic digesters, current operating conditions place significant constraints on their effectiveness. Although anaerobic digesters have longer sludge residence times than aerobic reactors, these periods remain too short, given the high wastewater throughput and the extremely low concentrations of MPs, to enable meaningful in situ biodegradation. The limited frequency of contact between MPs and enzymes or degradative microbes, combined with the continuous flow-through nature of the system, means that endogenous biological activity is unlikely to substantially reduce MP loads.

Consequently, any viable bio-based remediation strategy would need to act on MPs after they have been captured into more concentrated waste streams rather than within the bulk wastewater. MPs can pass through into the treated effluent and accumulate within the sludge, enabling their continued dispersal in aquatic and terrestrial environments, particularly when sludge is applied to soils as fertiliser. However, the rising presence of MPs in sludge could ultimately make it unsuitable for agricultural use^88^. For these reasons, sludge offers the most realistic intervention point, as MPs are substantially enriched in this fraction and retention times are comparatively longer. Even so, effective biological treatment would still require process redesign or dedicated configurations capable of sustaining contact between MPs and degradative agents, supporting adequate retention and managing biosafety and governance considerations tied to deploying engineered organisms in open treatment environments^89^.

Approaches employing immobilised enzymes or engineered plastic-degrading bacteria may therefore be viable, but only if the surrounding process architecture is specifically adapted to prevent washout and maintain stable operation. Thus, while bio-based remediation technologies could eventually be integrated into WWTPs, achieving this without major new infrastructure would require significant optimisation and thoughtful redesign of existing treatment stages^90^.

Additionally, any engineered strains deployed in such systems would be genetically modified organisms (GMOs) and therefore subject to strict containment regulations; their use would need to remain confined to closed WWTP environments and cannot involve release into natural ecosystems. Candidate organisms with relevant enzymatic activities include the aerobes *Thermobifida fusc*a and *P. sakaiensis* and two *Clostridium* species as anaerobic candidates capable of hydrolysing polyester^91^ or PET^92^. Should microbial PET degradation release intermediate products such as TA rather than achieve full mineralisation, these soluble compounds would need to be captured and treated within existing WWTP processes to prevent their discharge. Enzyme-only approaches may therefore offer a safer and more predictable route. Alternatively, organisms could be engineered not only to hydrolyse plastic substrates but also to assimilate the released monomers, a strategy that has shown early promise in engineered *Pseudomonas* strains for TA and EG utilisation^44,93,94^.

#### Implementation of biodegradation technologies in WWTPs

The potential use of GMOs requires further reflection about the potential impact of plastic-degrading bacteria on the activity of existing microbial communities within aeration tanks and anaerobic digesters, as well as about the ability of any introduced organisms to colonise, compete and persist in these systems. These concerns are amplified by the possibility of accidental release of engineered strains into the wider environment, a risk that carries regulatory, ecological and public-trust implications. Addressing these issues will require robust mitigation strategies. Some are already available within current WWTP practice, such as thermal hydrolysis, which sterilises sludge through high-temperature treatment. Others rely on advances in engineering biocontainment, ensuring that modified bacteria cannot survive outside tightly defined process conditions. Several stakeholders also noted that an alternative approach would be to forgo whole-cell systems entirely and use purified or immobilised plastic-degrading enzymes thereby removing any concerns related to microbial persistence or unintended release.

Research into the use of biotechnological techniques to remove MPs from aquatic environments is already underway. For example, the completed EU project Enzycle investigated enzyme-based strategies for microplastic degradation within WWTPs^95^. Similar efforts are also being pursued through other academic industrial partnerships and the previously mentioned programmes such as BOTTLE and the P3EB mission hub. While these biotechnological methods represent an exciting direction for microplastic mitigation, stakeholder discussions emphasised that their deployment must be accompanied by robust containment strategies, biosafety safeguards and transparent communication to ensure alignment with RRI principles.

### The challenge of operating at scale

Given the magnitude of the plastic problem, one key aspect of a bio-based responsible innovation is the challenge of its industrial scalability. These technologies are perceived to be in their infancy and operate slower than mechanical and chemical recycling, making their ability to process large amounts of plastic waste and generate high yields of the desired product a challenging feat. Consequently, bio-based methods were seen as complementary rather than an alternative to more established physicochemical technologies.

As with any new process, biological recycling or upcycling of plastic waste faces challenges, many of which have been reviewed by numerous authors^5–7,11,64,96,97^. While much can be done to improve the process, existing biological treatments already operate at industrial scale, with the enzymatic hydrolysis of PET being a great example. This has been achieved through, (i) the optimisation of reaction conditions and reactor design, (ii) substrate pre-treatment (increasing the susceptibility of crystalline structures to biodegradation), (iii) recovery and purification of products and (iv) the engineering of enzymes such as LCC^26^, Fast-PETase^37^ and PelB-LCC^12^ for greater degradation efficiency.

In the previously mentioned Carbios process, enzymatic depolymerisation of PET waste and polyester-textile materials yields monomers which, when sufficiently recovered, can be used to produce rPET of virgin-equivalent quality^67,98,99^. TA purification is relatively simple and efficient, whereas EG recovery requires energy-intensive distillation, which remains a cost- and emissions-relevant challenge for enzymatic recycling at scale. Following the operation of its demonstration plant opened in September 2021, Carbios has announced its first commercial facility in Longlaville, France, designed to process ~50,000 tonnes of prepared PET scrap per year and currently aiming for commissioning in the second half of 2027, subject to full funding and licensing^100^.

Carbios has also developed an extensive intellectual property portfolio, comprising 59 patent families as of the end of 2024 (https://www.carbios.com/en/enzymes/), covering areas such as enzyme engineering, enzymatic recycling processes, monomer recovery strategies and the production of biodegradable plastics. In parallel, recent academic work, including that of Murphy et al., has demonstrated additional opportunities to improve the sustainability and cost-effectiveness of enzymatic PET recycling^101^. For example, replacing sodium hydroxide with ammonium hydroxide during depolymerisation and neutralisation can facilitate recovery of the base and reduce chemical consumption and wastewater burdens, offering a potentially lower-impact alternative to conventional neutralisation approaches. Inspired by this success, other biological treatment concepts are being optimised to operate at scale. However, large-scale deployment remains contingent on techno-economic performance, feedstock availability and robust regulation.

#### The environmental footprint at scale

Our sustained engagement also prompted to consider the environmental footprint of bio-based technologies at scale. The energy and water consumption of biotechnological recycling processes compared with existing and developing technologies is an active subject of investigation^98^. A key challenge is the high-energy pre-processing required to overcome PET crystallinity, even in advanced industrial concepts such as those under development by Carbios. Commercial PET typically contains a large crystalline fraction, which is poorly accessible to enzymes and therefore needs to be amorphised, comminuted, or otherwise pre-treated before efficient depolymerisation can occur. These pre-treatments (e.g. extrusion, milling, amorphisation) contribute significantly to the overall energy demand of enzymatic recycling and remain a major target for process optimisation. This issue is particularly important for environmental applications, as plastics encountered in WW treatment facilities and in the wider environment often exhibit higher crystallinity and more complex morphologies than standard bottle-grade PET. This constraint is likely to be even greater for other polymers with higher glass transition temperatures or melting points (such as many polyamides and polyurethanes), for which operating at or near the glass transition temperature is less feasible at scale.

Nevertheless, biodegradation of plastic waste such as PET can occur at temperatures ranging between 40 and 70 °C^92^, a much lower range than that required for thermal and many chemical recycling methods. For example, PET pyrolysis requires temperatures around 500 °C while PET hydrolysis, glycolysis and methanolysis typically operate between 80 and 250 °C^102^. However, biological recycling of PET requires other energy inputs.

Recent modelling and life cycle assessment (LCA) studies^67^ indicate that for enzymatic PET hydrolysis to compete with virgin PET production and other closed-loop recycling technologies, electricity consumption must be substantially reduced, with amorphisation and reactor operation identified as particularly energy-intensive steps. Since the most energy-intensive step is often associated with overcoming crystallinity^103^, this could be mitigated by developing more energy-efficient feedstock pre-treatment techniques^104–109^, as opposed to using extrusion or cryo-milling methods, or by engineering enzymes that more effectively attack semi-crystalline PET^98^. In parallel, there is active work on protein engineering to improve performance under process-relevant conditions^13,110,111^, including enhanced thermostability^112–114^, tolerance to higher substrate loadings^103,115^ and operation at lower pH to reduce the need for continuous base addition for pH control^116^.

In addition to electricity consumption, current process designs for enzymatic PET recycling also require steam for EG recovery, sodium hydroxide for pH control of the depolymerisation reactor and cooling water, all of which are energy intensive. This provides a process optimisation opportunity for improving the sustainability of enzymatic PET hydrolysis against other recycling technologies^88,89^. Moreover, the logistics of enzyme supply are a central consideration.

Although large-scale enzyme production and purification can be energy and resource intensive, recent process concepts typically employ low enzyme loadings and often rely on crude or partially purified preparations rather than highly purified enzymes, reducing some of the associated burdens. Industrial-scale recombinant protein production is already widely established, for example, in the manufacture of cellulases for second-generation bioethanol, proteases and lipases for detergents and a range of food and feed enzymes, demonstrating that multi-kilotonne annual enzyme outputs are technically feasible.

However, perspective is important: even at an optimised loading of ~1 mg of enzyme per gram of PET, the quantities required become substantial when scaled to real industrial throughputs. Treating 1 tonne of PET would require around 1 kilogram of enzyme, and treating 100,000 tonnes, a typical capacity for a regional PET recycling plant, would require ~100 tonnes of enzyme per year. These values are within the order of magnitude of today’s industrial enzyme markets but would still represent a significant dedicated production capacity if enzymatic recycling were deployed broadly. For this reason, the relative merits of supplying enzymes directly versus employing whole-cell microbial processes remain system-dependent, and neither option is universally more energy efficient. In other industrial biotechnology sectors such as second-generation bioethanol production, on-site enzyme production has been used to avoid energy-intensive concentration and shipping of enzyme formulations, and similar strategies could be envisaged for enzymatic plastic recycling. Whether whole-cell microbial processes would ultimately be less energy demanding than enzyme-based processes therefore remains an open question and is likely to be highly system-dependent, rather than universally true.

The research cited above answers some of the calls for comprehensive LCA studies and assessments of upstream pre-treatments, downstream monomer recovery and purification, WW treatment or reuse^5,10^, disposal of waste by-products and technoeconomic analyses (TEA)^5,8^. These analyses are essential if this technology is to progress to an industrial scale^5^. The planned construction and deployment of Carbios’ PET depolymerisation plant demonstrates that enzymatic PET recycling has reached an advanced TRL and is considered commercially promising. However, the decision to scale up does not prove that LCA and TEA outcomes are unequivocally favourable, as large-scale projects can also be driven by policy incentives and subsidies and may still face economic uncertainties. Ongoing independent LCA and TEA studies will therefore be crucial to determine under which conditions enzymatic PET recycling can deliver robust environmental and economic benefits compared with both virgin PET production and alternative recycling technologies. Beyond process performance, questions surrounding the nature, use and disposal of end-products introduce an additional layer of societal and environmental consideration.

### End product and waste disposal

As in any biotechnological process, downstream processing and purification of the final products are factors that greatly contribute to the feasibility of plastic upcycling. High titres and purity are essential in any biotransformation, and any end-products must adhere to strict specifications and quality standards which depend on the final application. In this regard, several of the projects investigating plastics as a substrate for biotechnology focus on the generation of novel and biodegradable materials. Special consideration needs to be given to the fate of such products at the end of their functional life: whether they can themselves be recycled, whether they introduce new waste-management challenges, and how their life-cycle impacts compare to conventional plastics. These concerns must be considered critically, as they highlight the importance of ensuring that bio-based innovations do not unintentionally generate new waste burdens or shift environmental impacts downstream.

There is no simple answer, given the diversity of products that could be generated taking advantage of microbial metabolic processes. Some monomers derived from plastic biodegradation and biotransformation may be used to create non-plastic products that have easier routes for recycling or indeed may not need recycling. For example, as mentioned previously, the PET monomer TA can be upcycled into the small value-added molecule vanillin^42^. However, it must be noted that the global market for vanillin, or for other fine chemicals such as paracetamol^45^, is orders of magnitude smaller than global PET waste volumes. Therefore, such transformations should be viewed not as primary outlets for large-scale PET depolymerisation but as high-value side-streams within a broader biorefinery model, in which the bulk of monomers would be recycled back into PET while a fraction could be diverted to higher-margin products.

While such high-value transformations provide useful outlets for a fraction of recovered monomers, most depolymerised material will inevitably be directed back into the synthesis of new plastics. This makes their end-of-life fate a central consideration and raises questions about whether these pathways introduce new circularity challenges or genuinely contribute to closing material loops. Examples of this include the direct recycling of TA and EG into fresh PET; the biosynthesis of adipic acid^117^ or hydroxyalkanoic acids^48^, which can serve as building blocks for other polymers such as Bio-PU; and the biosynthesis of polyhydroxyalkanoates (PHAs) from TA^47,118^. These bioplastics are of microbial origin and are, in principle, biodegradable. However, these reflections underscore that biodegradability is highly context-dependent: even plastics commonly described as biodegradable, such as PLA and polycaprolactone, can be recalcitrant depending on formulation^119^ and many nominally biodegradable plastics require industrial composting conditions to degrade efficiently. Moreover, while biodegradability is essential in applications involving deliberate environmental release, such as agricultural mulching films or controlled-release products, in most other use cases, recycling rather than environmental degradation remains the preferred end-of-life pathway. Together, these considerations highlight the need to prioritise robust design-for-recycling principles alongside biodegradability when developing new bio-based polymers.

### Economic, policy and societal matters

**Fig. 3:**
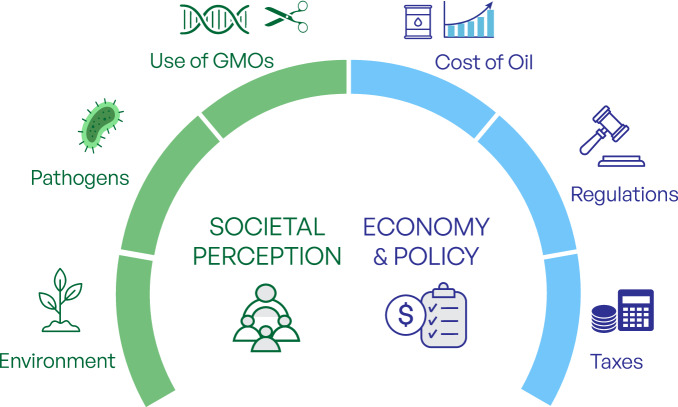
Topics of interest around societal implications, economics and policy making. Bio-based technologies are perceived as having a beneficial environmental effect, but there are concerns about the potential contamination of food-grade plastics with microbial pathogens and the accidental release of genetically modified organisms (GMOs). The adoption of any new plastic management technologies would be conditioned by the cost of virgin plastics, the implementation of regulations limiting their use and taxation.

Our discussions also reflected on economic and policy matters relating to the biodegradation of plastic waste, with energy and scale-up costs highlighted as important considerations, illustrated in Fig. 3. The recent hike in global energy prices, particularly for grid electricity in many regions, has increased attention on the energy demands of novel recycling processes. However, it is important to acknowledge that renewable electricity, especially large-scale solar and wind, has reached extremely low costs in certain regions (e.g. <1.5 € cent kWh⁻¹ in Chile and Saudi Arabia), whereas plastic-recycling facilities must operate where collected waste is located, often in regions with significantly higher electricity prices. These geographical disparities underscore the need for TEAs and LCAs^67^ to evaluate site-specific impacts and to identify where energy efficiencies or renewable integration would yield the greatest benefit.

It is widely accepted that the largest challenge for adoption of bio-based plastic waste management is its cost^102^. Costs are associated with industrial-scale fermenters, enzyme production and the downstream energy demands of depolymerisation and monomer recovery. Although several engineered PET hydrolases, including high-performance LCC variants and signal-peptide-optimised PETases^94,120–124^, can be efficiently secreted by microbial hosts without the need for laborious purification, the major costs of biological recycling still arise elsewhere, particularly in feedstock preparation and reactor operation. Another significant cost relates to monomer recovery where TA is obtained through acidification and precipitation, a comparatively straightforward step but one that still requires chemical inputs, pH-adjustment reagents, filtration or centrifugation equipment and handling of the resulting salt streams. In contrast, recovering EG typically depends on energy-intensive distillation, demanding substantial thermal energy and capital-intensive separation equipment. Therefore, scale-up and commercialisation of biological recycling methods will involve considerable investment from both industry and governments.

Ideally, investment in these technologies would lead to the downstream production of bio- or recycled plastics in a cradle-to-cradle approach. Carbios and partners are investing in the world’s first PET bio-recycling plant, the cost of which was re-estimated at €230 million in 2023^125^. With such investment, it is imperative that there are clear and stable markets for the end products of plastic biodegradation. In the case of PET, new markets need to be broadened for its monomers, EG and TA. As illustrated in Fig. 1, these can be used to manufacture PET (rPET) or transformed into high value chemicals for use in the manufacture of other products such as Bio-PU, thus maximising the economic potential of biological recycling and upcycling.

However, modelling studies suggest that the minimum selling price (MSP $/kg) of rPET derived from enzymatic biodegradation technologies is higher than that of rPET from mechanical or chemical recycling and higher than virgin PET from petroleum^67^. This raises the question of whether manufacturers would be prepared to pay more for polyols, such as EG, derived from biodegradation processes, or whether they would default to cheaper oil-derived substrates. Our general impression is that many manufacturers would choose the less expensive option. As others have proposed, plastic may need to be decoupled from fossil-fuel pricing to create a stable market for recycled materials^126^, preventing fluctuations in oil price from undermining demand for rPET and other recycled polymers. The lack of a separate demand for recycled plastic material independent of virgin plastic has historically constrained recyclables markets^127^. Nevertheless, market conditions in Europe have begun to shift: several major brand owners, not just traditional sustainability-driven businesses like outdoor and apparel, have set ambitious rPET content targets and advanced into large-scale applications, including food-packaging and detergent formats. Increasing rPET uptake in several applications is also being driven by EU regulatory measures, including recycled-content requirements and EPR frameworks. This trend is reflected in increasing rPET collection, investment in capacity and converging pressures from policy, consumers and corporate commitments^128–130^. However, despite this momentum, market demand remains sensitive to fluctuations in quality, supply and pricing, meaning supportive policy frameworks are essential to sustain and stabilise progress.

Therefore, policies are needed to create stable, long-term markets for materials derived from recycling processes and to address supply, consistency and price^131^. Policies such as EPR schemes can ensure both the supply of plastic waste for recycling and the inclusion of mandatory proportions of recycled materials in products^132^. For example, the EU Directive 2019/904 requires that by 2025 and 2030, 25% and 30% of PET bottles manufactured in EU Member States must be rPET. The UK is also rolling out its own EPR reforms for packaging^63^, that began in 2025, which will place the full net cost of managing packaging waste onto producers and is expected to increase the availability and quality of collected recyclate^133^. Complementing EPR, the UK government has committed to a unified Deposit Return Scheme (DRS) covering England, Northern Ireland and Scotland, with regulations that came into force in January 2025 and a launch date set for 1st October 2027^134,135^. This approach aligns with the success of DRS implementations across several European countries, such as Germany, Denmark, Romania and the Netherlands, which have achieved consistently high return rates (>90 %) for beverage containers and secured high-quality PET and aluminium feedstocks for recycling.

In addition, regulatory mechanisms will be needed to ensure that monomers derived from biodegradation processes, such as TA and EG from PET, can be accurately traced to the recycled content of products, validating any claimed green credentials. In this regard, taxation can play a role regulating prices by providing clear economic incentives to increase the use of recycled materials. For example, since April 2022, the UK has implemented the Plastic Packaging Tax (PPT), levied on packaging that does not contain at least 30% recycled plastic (£223.69 per tonne from April 2025). Taxation may also target raw materials used for manufacturing plastic products, imposing higher taxes on primary or non-sustainable feedstocks while subsidising recycled or more sustainable alternatives^136^. Together, these regulatory and fiscal measures, including EPR, DRS and PPT, can help stabilise markets for recycled materials, reduce dependence on virgin plastics and create the economic conditions needed for biological recycling technologies to scale successfully.

### Societal attitudes towards plastic biodegradation

So far, we have considered technical and economic aspects of plastic biodegradation as well as alternative applications. However, engagement activities highlighted the need to pay attention to societal responses to this technology, given that concerns exist about the use of GMOs and the potentially negative connotations associated with microbes following the COVID-19 pandemic^137–139^. Biotechnology and the strong messages associated with its application for tackling plastic waste need to be communicated to the public with transparency, clearly explaining its limitations and improvements. Creative and arts-based engagement approaches have recently been shown to enhance public understanding of enzyme science while fostering more meaningful^140^, two-way dialogue about biotechnology, particularly by making complex research accessible to diverse non-academic audiences.

Recent social science research showed that if GMOs are discussed in concrete language with specific applications (rather than in abstract language), public perceptions tend to be more positive towards these applications and genetic modification in general^137^. The results from a large survey conducted by Commonwealth Scientific and Industrial Research Organisation (CSIRO) using 7 synthetic biology applications challenged the common view of generalised public opposition to genetic modification technologies^141^. Furthermore, a recent survey by Carbios found that consumers saw enzyme-based technologies as a promising solution to help solve the plastic pollution problem^142^.

Across our engagement activities, we noticed a broad support for the use of biotechnology to address the plastic waste crisis. The urgency of plastic pollution was frequently perceived as outweighing reservations about biotechnology itself. At the same time, in our activities we have faced recurring concerns regarding the potential leakage or mismanagement of plastic-degrading microbes and the hypothetical risk of unintended impacts on non-waste plastics. To minimise such risks, researchers including us argue about the importance of ensuring that any biodegradation processes would operate within safety frameworks comparable to those governing existing industrial biotechnological applications. Moreover, microbes are unlikely, or could be engineered not, to survive outside controlled reactor conditions, reinforcing the perception that most risks would be governed through design rather than behavioural controls. Another area of concern is the use of biodegradation-derived products in food-contact applications, highlighting the need for robust technological design, clear regulatory oversight and transparent safety assurance where microbial systems form part of closed industrial processes rather than components of final consumer products.

### Concluding remarks

Addressing the extensive nature of the plastic waste crisis warrants a range of recycling and upcycling technologies, of which biotechnology, spanning both enzymology and microbiology, is only one component. This RRI-informed perspective suggests that stakeholders and the wider community are broadly supportive of these technologies but at the same time, recognise the many challenges still to be overcome, such as the need for transparent communication, clearer evidence of environmental safety and realistic expectations around scalability and applicability. These insights highlight not only the scientific challenges ahead but also the importance of engaging with societal perspectives early in the innovation process.

Despite the challenges outlined above, biotechnological approaches have already progressed to pilot and early industrial deployment, most notably for the recycling of polyester-based plastics including textile-derived PET. These developments demonstrate the potential contribution of biotechnology to emerging circular-economy models for plastics. Moreover, biotechnological tools may, in the longer term, extend beyond recycling and upcycling to applications in bioremediation, for example in the removal of MPs or legacy additives from sewage sludge before land application.

Substantial advances have been made in the biodegradation, recycling and upcycling of plastic waste in recent years. These reflections, however, underscore that scientific progress alone will be insufficient if it is not accompanied by responsive, participatory and anticipatory research practices. Integrating societal concerns into the evolution of these technologies, whether relating to biosafety, feedstock availability, infrastructural integration or environmental impact, is not a peripheral consideration but a central condition for legitimacy and long-term success. An RRI-informed approach offers a structured means of aligning emerging biotechnological solutions with societal expectations, enhancing both trust and relevance. With sustained collaboration across academia, industry, policymakers and an informed public, biotechnological approaches are well positioned to contribute meaningfully to more responsible and resilient circular-economy models for plastics.

## Supplementary information

Supplementary Information
