# Supplementary material for: Reflections on bio-based PET and plastic waste management: a responsible research and innovation approach
Source: Nat Commun. 2026 Mar 6;17:2281. doi: 10.1038/s41467-026-69970-4 (PMC12972147; doi:10.1038/s41467-026-69970-4)
Supplement: Supplementary file 1 — Supplementary Information [file 41467_2026_69970_MOESM1_ESM.pdf]

*Supplementary information for*

# Reflections on Bio-based PET and Plastic Waste Management: a Responsible Research and Innovation Approach

Joanne Benton[1], Catalina Cruañas-Paniker[1,2], Brooke Wain[1,3] and José I. Jiménez[1,3*]

[1]Department of Life Sciences, Imperial College London, London, SW7 2AZ, United Kingdom

[2]Science and Solutions for a Changing Planet DTP, Imperial College London, London, SW7 2AZ, United Kingdom

[3]Imperial Centre for Engineering Biology, London, SW7 2AZ, United Kingdom

[*]Correspondence to j.jimenez@imperial.ac.uk

## Note 1: Ethics and consent

The engagement activities described in this Perspective were conducted to inform project communication and the authors' reflections on Responsible Research and Innovation (RRI). Stakeholders and public attendees were informed about the purpose of the engagement and provided informed consent for notetaking and for the use of anonymised, non-identifiable insights in publications. No personally identifying information was collected or reported, and no verbatim quotations are included. Engagement with stakeholders for the purpose of evaluating the author's research activities does not require ethical approval according to institutional guidelines.

## Note 2: Stakeholder engagement activities

Stakeholder and public engagement activities took place between June 2021 and February 2025. These activities included semi-structured conversations with 26 stakeholders representing key points across the plastics value chain, including industry (waste management, wastewater, PE, PP, PU and PLA manufacturing, mechanical and chemical recycling; n = 13), recycling and social enterprises / NGOs (n = 5), and academia (n = 8).

Stakeholders were invited to engage based on their professional roles within the plastic value chain, their involvement in sustainability and recycling, and their interest in the broader implications of biotechnological approaches to plastic waste management. Contacts were identified through professional networks, publicly available organisational information, and recommendations arising

through engagement. Invitations were issued by email together with a project storyboard outlining the scientific basis, scope and aims of the MIPLACE project (Supplementary Note 6). Conversations were semi-structured and typically lasted 30-60 minutes. This format was chosen because it supports the exploration of different perspectives while maintaining enough structure to allow common issues to be discussed across engagements (2). Open-ended prompts were used to encourage contributors to elaborate on topics they considered most relevant, helping to surface a range of viewpoints and considerations (3).

Before each conversation, contributors were informed of the purpose of the engagement activities and gave permission for anonymised notes to be used to inform project communication and academic publications. No personal data or identifying information were collected. Notes taken during and after conversations were reviewed by the author team to identify recurring themes that informed the reflections presented in the manuscript. This approach supported the exploration of diverse perspectives and provided insight into stakeholder experiences and expectations (2).

**Note 3: Stakeholder conversation prompts**

The following prompts were used to guide stakeholder conversations and to support open discussion during engagement activities.

1. What are your overall thoughts on the MIPLACE approach for tackling plastic waste? Discuss positive and negative aspects.
2. Are there any aspects of this research that you would like to know more about?
3. Is there anything else that you would like the technology to do?
4. Do you have any thoughts on an LCA for the process – what would you consider to be key considerations?
5. Is there the scope to supply PET/PU for this method of recycling as well as for other recycling technologies?
6.
    a. Generally, how do you think the PET/PU industry would feel about using products that have been manufactured in part using components from bioprocesses?
    b. Bioplastic production may be a more expensive process than conventional methods. Do you think that industries would be willing to pay more for more sustainable products that are made in part by bioprocesses.

7. On a more general note, the more 'sustainable' products generally tend to be more expensive. What is your opinion on this?

8. Do you think the technology is socially desirable and in the public interest?

9. Does terminology influence how you think about the research? For example, the use of the term GMO as opposed to something like 'engineered organisms'.

10. How do you think this research is best communicated to wider audiences to gain approval from those who may be less certain about the use of this technology?

11. Overall, would you accept (or approve of) this technology being a contributor to the mitigation of plastic (PET and PU) waste? (For this question, the 5-point Likert scale can be used where 1 is strongly disapprove, 2 is disapprove, 3 is neutral, 4 is approve and 5 is strongly approve) (1).

    Please can you give a reason for your answer.

12. Finally, would you like to make any further comments?

**Note 4: Public engagement activities**

In addition to stakeholder engagement described above, we also undertook public-facing engagement activities to explore how bio-based plastic technologies are perceived beyond professional audiences. A two-day interactive workshop on microbial plastic degradation was delivered at the Great Exhibition Road Festival in June 2022 to children aged 5-12 and their families, and an interactive presentation for adult community members (50+) from three London boroughs was delivered in September 2022.

At both events, attendees were invited to respond (yes/no/unsure) to attitudinal statements: *Does our research benefit society? Are microbes breaking down plastic for use in new products a good idea? Would you pay more for bioplastic products? Will you use less plastic in future?* Attendees could also leave free-text comments. Observational notes and written responses were reviewed by the author team to identify recurring themes.

**Note 5: Scope and limitations**

Notes from stakeholder conversations and public engagement activities were reviewed by the author team to identify recurring concerns, expectations, and areas where clearer communication appeared to be needed. These themes were used to shape the reflections and discussion presented in the manuscript. No audio recordings were made.

The engagement activities involved a limited number of contributors and audiences and were not intended to provide statistically representative coverage of all stakeholder groups or the general population. Engagement with policymakers and school-aged groups would have provided additional perspectives but could not be secured within the project timeframe. Public engagement audiences reflect those who attended specific events rather than a demographically representative sample.

The purpose of these activities was therefore not to generate generalisable conclusions, but to support reflection on RRI considerations for emerging bio-based plastic management technologies and to highlight themes relevant to future research, governance and communication.

**Note 6: Storyboard**

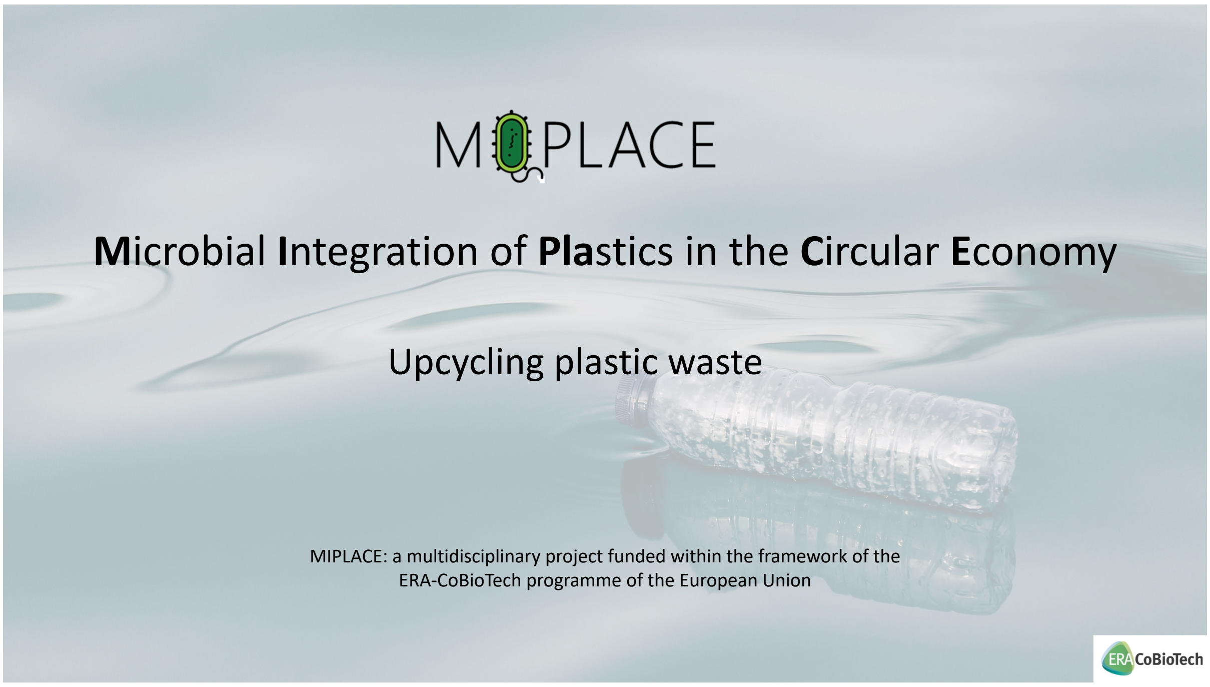

## Plastic waste: A global crisis

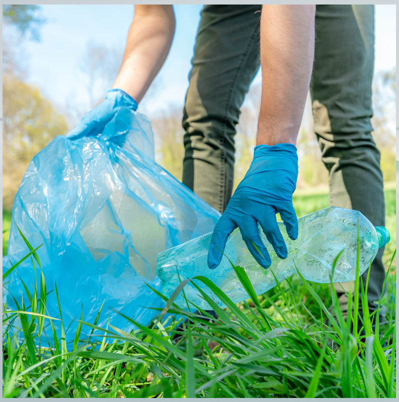

- 400 million tonnes of plastic produced globally each year
- Estimated 25% is incinerated and 56% going to landfill
- Global average recycling rates are 14-18%
- Plastic pollution poses environmental and health risks
- Use of fossil fuels for virgin plastics contributes to climate crisis

**Source: Plastics, the Circular Economy and Global Trade. World Economic Forum (2020)*

MIPLACE ERA CoBioTech

## Plastic recycling technologies

**Mechanical recycling**

- Plastic is sorted, washed and ground and the materials are recovered by remelting and regranulating.
- If the material is of good quality, the recycled materials can be converted into the same or similar type plastic products.
- However, the recycling process can result in 'downcycling' as high temperatures and shear forces can reduce the quality of the recycled material.
- There is a limit on how many times plastic can be recycled by this method.

**Chemical recycling**

- For recycling plastics that are mixed with other materials or different types of plastics.
- There are different types of chemical recycling processes such as glycolysis and hydrolysis.
- Plastic is broken down by chemical transformation into its building blocks and used to form new plastics or upcycled to higher value chemicals.
- Generally, no limit to the number of times plastic can be recycled.

*Sources: European Bioplastics: Mechanical recycling (July 2020); Zhu* et al *(2021) Enzyme discovery and engineering for sustainable plastic recycling. Trends in Biotechnology; Plastics, the Circular Economy and Global Trade. World Economic Forum (2020); Plastic pollution: how chemical recycling technology could help fix it. The Conversation (2021); Chemical Recycling Europe (2019); EEA Report No. 18/2020*

MIPLACE ERA CoBioTech

## The MIPLACE approach for upcycling PET and PU plastic waste

- MIPLACE **aims** to develop an efficient **bio-based process** that converts plastic waste (PET and PU) into molecules of value that can be **upcycled** into new products thus contributing to a **circular economy**.
- **Microbial communities** will **enzymatically** degrade PET and PU into their constituent monomers and transform them into building blocks for the synthesis of Bio-PU, a more environmentally-friendly construction and insulation material.
- Microbial degradation and transformation offers an additional approach alongside current plastic recycling technologies.
- Operating at lower temperatures, biodegradation methods may have lower energy inputs/costs and a reduced carbon footprint.

**How to create microbial communities to transform PET and PU waste into building blocks for Bio-PU?**

Screening of microorganisms from the environment for plastic degrading activity

Designing microbial strains for greater efficiency by applying synthetic biology techniques

Selecting for plastic degraders by adapted laboratory evolution (ALE)

*PET: polyethylene terephthalate commonly used for single-use plastics especially in the beverage industry*
*PU: polyurethane (foams) used in insulation panels, carpet underlay, furniture and bedding, footwear*

## Techniques in a nutshell

- **Synthetic biology:** employing engineering techniques to redesign organisms for beneficial applications such as solving problems within agriculture and the environment.
- Pieces of DNA from an organism, or novel DNA, are inserted into another organism's genome thus changing the genetic code and the activities of the recipient organism.

- **Adapted laboratory evolution (ALE):** the continuous culturing of individual strains or communities on PET or PU as the sole carbon source.
- As a result of such selection pressures, better plastic degraders will emerge.

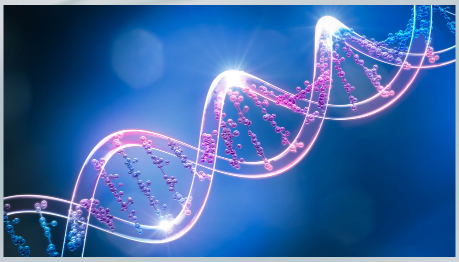

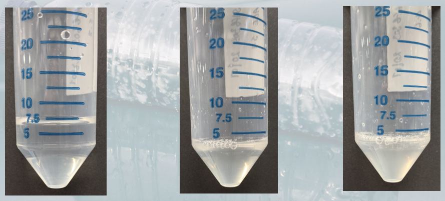

**Adapted laboratory evolution (ALE):** From left to right: increasing turbidity as a strain of *Pseudomonas putida* adapts to grow on terephthalic acid (TA). *Source: Dr. Alice Banks (ICL)*

## Upcycling PET and PU plastic waste: a more detailed look

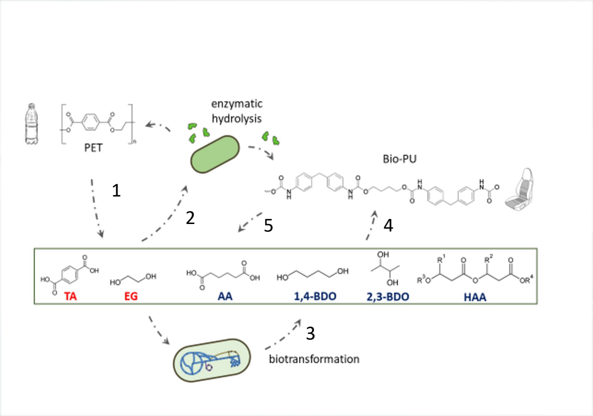


Microbes will perform enzymatic hydrolysis of PET and PU plastic waste to produce monomers (1). These monomers (red) support microbial growth (2) but also undergo biotransformation (3) into other monomers (blue) that may be used to synthesize Bio-PU (4) so achieving the upcycling of plastic waste.

Bio-PU is used as a construction and insulation material and can be recycled (5) at the end of its life demonstrating a **circular approach for tackling PET and PU waste.**

*PET = polyethylene terephthalate; PU = polyurethane; EG = ethylene glycol; TA = terephthalic acid;*
*AA = adipic acid; 1,4-BDO = 1,4-butanediol; 2,3-BDO = 2,3-butanediol; HAA = hydroxyalkanoyloxy-alkanoic acid*

MIPLACE ERA CoBioTech

## MIPLACE: Some technical details

**MIPLACE: Technology readiness levels (TRLs)**

**Aim:** microbial hydrolysis of PET and PU, production of monomers and synthesis of Bio-PU from plastic waste operating at industrial scales (TRL 5 or above)

**Current situation**

- Enzymatic hydrolysis of PET (TRL 4)
- Enzymatic hydrolysis of PU (TRL 4)
- Synthesis of Bio-PU from monomers (TRL 4)

**Technology readiness levels (TRLs)**

**TRL 3**: experimental proof of concept
**TRL 4**: technology validated in lab
**TRL 5**: technology validated in relevant environment (industrially relevant environment in the case of key enabling technologies)
**TRL 6**: technology demonstrated in relevant environment (industrially relevant environment in the case of key enabling technologies)

**Examples of enzyme activities**

- **PET hydrolase**: a minimum of 90% PET depolymerization into monomers over a 10-hour period. Mean productivity of 16.7g terephthalate per litre per hour (Tournier *et al*, 2020).
- **Polyester hydrolase, PHL7**: completely hydrolysed amorphous PET films, releasing 91 mg of terephthalic acid per hour and mg of enzyme (Sonnendecker *et al*, 2021)
- French company, **Carbios**, launched a demonstration plant for its enzymatic recycling technology in September 2021.

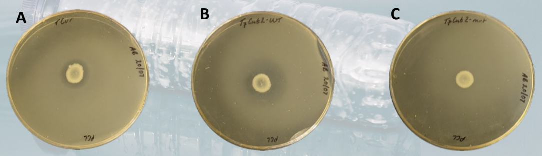


**Plates showing degradation of the polymer, polycaprolactone (PCL), a model substrate for plastic degradation**. Plates A and B show ‘halos’ surrounding microbial growth where the polymer has been degraded by enzyme activity. In Plate C, the halo is absent as the enzyme responsible for degradation is inactive. *Source: Dr. Alice Banks (ICL)*


*Source: Tournier et al (2020) An engineered PET depolymerase to break down and recycle plastic bottles. Nature, 580, 216-219.*
*Sonnendecker et al (2021) Low carbon footprint recycling of post-consumer PET plastic with a metagenomic polyester hydrolase. ChemSusChem 10.1002/cssc.202101062*
*Carbios press release 29 September 2021*


MIPLACE ERA CoBioTech

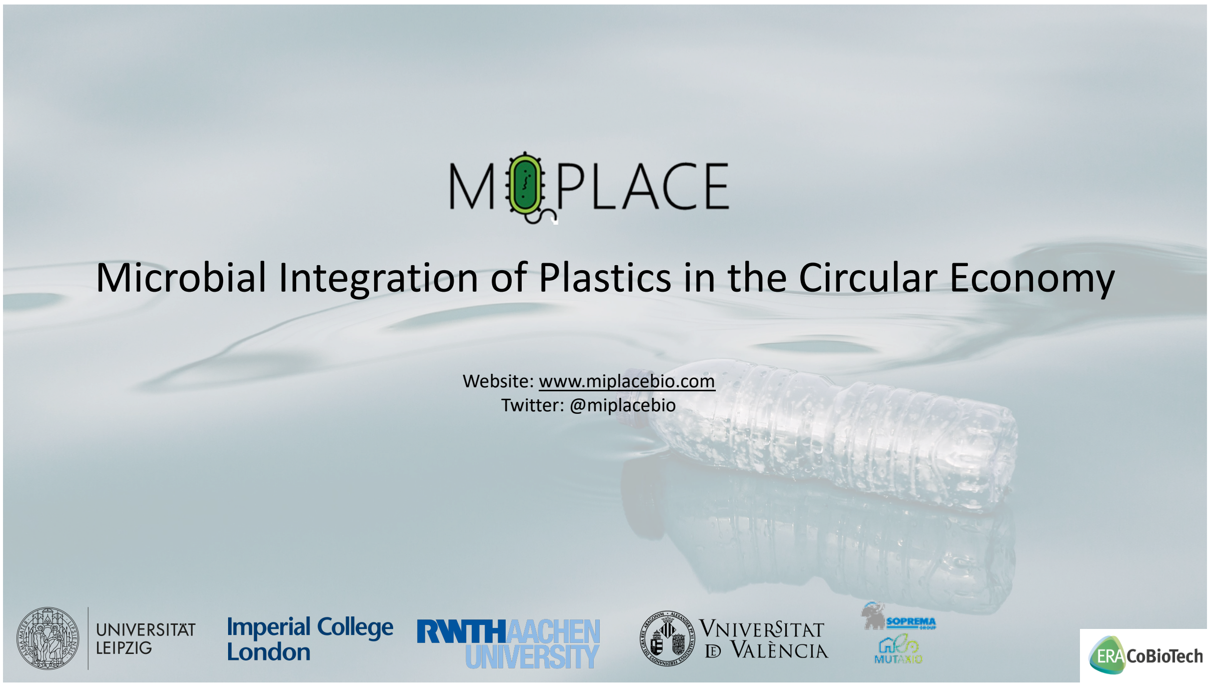


## Supplementary references


1. Filho WL, Salvia AL, Bonoli A, Saari UA, Voronova V, Klõga M, et al. An assessment of attitudes towards plastics and bioplastics in Europe. Science of The Total Environment. 2021 Feb 10;755:142732.

2. Evans C, Lewis J. Analysing semi-structured interviews using thematic analysis : exploring voluntary civic participation among adults. London: SAGE Publications Ltd.; 2018.

3. Brinkmann S, Kvale S. Doing Interviews [Internet]. Second. 55 City Road, London: SAGE Publications Ltd; 2025. Available from: https://methods.sagepub.com/book/mono/doing-interviews-2e/toc
